# Luminescent Hydrogel Based on Silver Nanocluster/Malic Acid and Its Composite Film for Highly Sensitive Detection of Fe^3+^

**DOI:** 10.3390/gels7040192

**Published:** 2021-10-31

**Authors:** Xiangkai Liu, Chunhui Li, Zhi Wang, Na Zhang, Ning Feng, Wenjuan Wang, Xia Xin

**Affiliations:** 1 Key Laboratory of Colloid and Interface Chemistry (Ministry of Education), School of Chemistry and Chemical Engineering, Shandong University, Jinan 250100, China; lxk575730669@163.com (X.L.); li_chunhui0411@163.com (C.L.); zwang@mail.sdu.edu.cn (Z.W.); 202020302@mail.sdu.edu.cn (N.F.); wenjuanwang@mail.sdu.edu.cn (W.W.); 2 China Research Institute of Daily Chemistry Co., Ltd., Taiyuan 030001, China

**Keywords:** silver nanoclusters, malic acid, self-assembly, AIE, sensor

## Abstract

Metal nanoclusters (NCs) with excellent photoluminescence properties are an emerging functional material that have rich physical and chemical properties and broad application prospects. However, it is a challenging problem to construct such materials into complex ordered aggregates and cause aggregation-induced emission (AIE). In this article, we use the supramolecular self-assembly strategy to regulate a water-soluble, atomically precise Ag NCs (NH_4_)_9_[Ag_9_(C_7_H_4_SO_2_)_9_] (Ag_9_-NCs, [Ag_9_(mba)_9_], H_2_mba = 2-mercaptobenzoic acid) and L-malic acid (L–MA) to form a phosphorescent hydrogel with stable and bright luminescence, which is ascribed to AIE phenomenon. In this process, the AIE of Ag_9_-NCs could be attributed to the non-covalent interactions between L–MA and Ag_9_-NCs, which restrict the intramolecular vibration and rotation of ligands on the periphery of Ag_9_-NCs, thus inhibiting the ligand-related, non-radiative excited state relaxation and promoting radiation energy transfer. In addition, the fluorescent Ag_9_-NCs/L–MA xerogel was introduced into polymethylmethacrylate (PMMA) to form an excellently fluorescent film for sensing of Fe^3+^. Ag_9_-NCs/L–MA/PMMA film exhibits an excellent ability to recognize Fe^3+^ ion with high selectivity and a low detection limit of 0.3 μM. This research enriches self-assembly system for enhancing the AIE of metal NCs, and the prepared hybrid films will become good candidates for optical materials.

## 1. Introduction

Metal nanoclusters (NCs), such as gold, silver, and copper NCs, represent a class of multifunctional materials with attractive optoelectronic and photoluminescence properties [1,2,3,4,5]. It consists of a metal core, composed of several to hundreds of metal atoms and a peripheral organic ligand, forming a unique core-shell structure [6,7,8,9,10]. Due to their large Stokes shift, low toxicity, good biocompatibility, and other excellent characteristics, metal NCs can be used as environmentally friendly and biocompatible color conversion materials, fluorescent probes, and excellent biological probe for protein expression [11,12,13,14,15,16,17,18,19].

Recently, the aggregation-induced emission (AIE) strategy has been used to enhance the photoluminescence of metal NCs, thereby enhancing its application in fluorescent sensing, light-emitting diodes, and other optoelectronic devices [20,21,22]. At present, the commonly used methods for AIE are solvent and cation-induced aggregation. However, these two methods cannot obtain ordered aggregates, resulting in instability and poor uniformity of nanocluster aggregation [23,24,25,26]. Therefore, an effective strategy for manipulating the spatial arrangement of nanostructured units to form a specific structure: self-assembly, was introduced to solve this problem. By regulating the non-covalent forces between multiple molecules (van der Waals forces, hydrogen bonds, electrostatic interactions, and π-π stacking), metal NCs, and other molecules form ordered aggregates with specific functions, which further improves the photoluminescence performance of the metal NCs [27,28,29]. For example, Shen et al. used the supramolecular self-assembly strategy to obtain stable colloidal aggregates (nanospheres and nanovesicles) of Ag_6_-NCs/PEI, through multiple electrostatic interactions between Ag_6_-NCs and polyethyleneimine (PEI) [30]. During the formation of the order structure, the Ag_6_ NCs luminescence in the dilute aqueous solution was turned on. Zhang et al. demonstrated the enhancement of the luminescence of Cu NCs by forming compact and ordered self-assembly architectures by changing the annealing temperature in the formation of Cu NCs [31]. Therefore, it can be seen that the self-assembly of metal NCs is very attractive and worth studying.

Fluorescent film sensors are widely considered, due to their advantages, such as convenient portability, real-time detection, and no pollution to the system, for testing [32,33,34,35,36,37]. Compared with the solution and powder detection forms, the adjustable shape, size, and flexibility of the fluorescent film makes it have wider application prospects. For example, Li et al. optimized the concentration of octadecylamine with amino groups in n-hexane to make the Tris-base modified silver NCs self-assemble into a single-layer silver nanocomposite film at the n-hexane-water interface and use it as a surface enhancement Raman scattering (SERS) active substrate for ultra-sensitive detection of Hg^2+^ ions [38]. The SERS sensing monolayer film also has good reproducibility and recovery rate. Katowah et al. used an in-situ method to prepare a ternary nanocomposite film containing copper oxide/polymethylmethacrylate (PMMA)/various carbon-based nanofillers, as a selective Hg^2+^ ion sensor and the mixed nanofiller significantly improved performance of PMMA film [39].

**Scheme 1:**
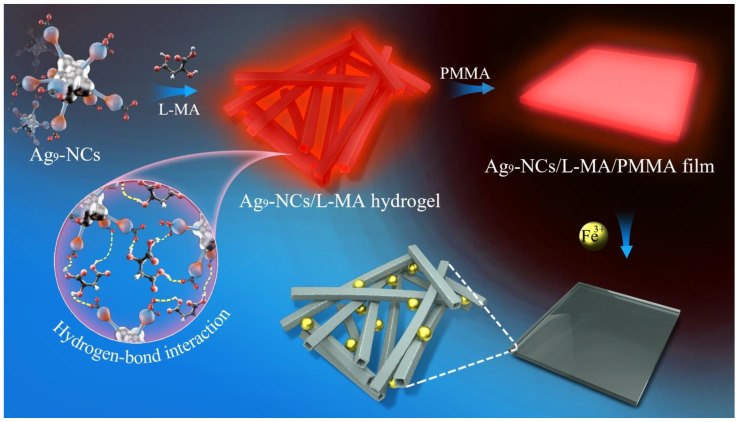
Schematic illustration of the hollow-tube structure of Ag_9_-NCs/L–MA hydrogel and its composite film for highly sensitive detection of Fe^3+^.

Herein, a water-soluble, atomically precise Ag NCs (NH_4_)_9_[Ag_9_(C_7_H_4_SO_2_)_9_] (Ag_9_-NCs, [Ag_9_(mba)_9_], H_2_mba = 2-mercaptobenzoic acid, and the molecular structure of Ag_9_-NCs is shown in Figure S1) were selected to self-assembled with L-malic acid (L–MA) to construct phosphorescent hydrogels (Scheme 1). The Ag_9_-NCs/L–MA hydrogel has a hollow-tube structure which is regulated by non-covalent interactions, based on hydrogen bonds, which further promotes the AIE phenomenon. In order to further develop its application prospects as fluorescent sensors, Ag_9_-NCs/L–MA xerogels are doped into PMMA films to construct fluorescent film sensors for detecting Fe^3+^ ions. The value of the corresponding quenching coefficient, K_SV,_ is 5.2 × 10^4^ M^−1^ in the low concentration range, and the detection limit of Fe^3+^ is down to 0.3 μM.

## 2. Results and Discussion

### 2.1. Self-Assembly of Ag_9_-NCs/L–MA Hydrogels

**Figure 1:**
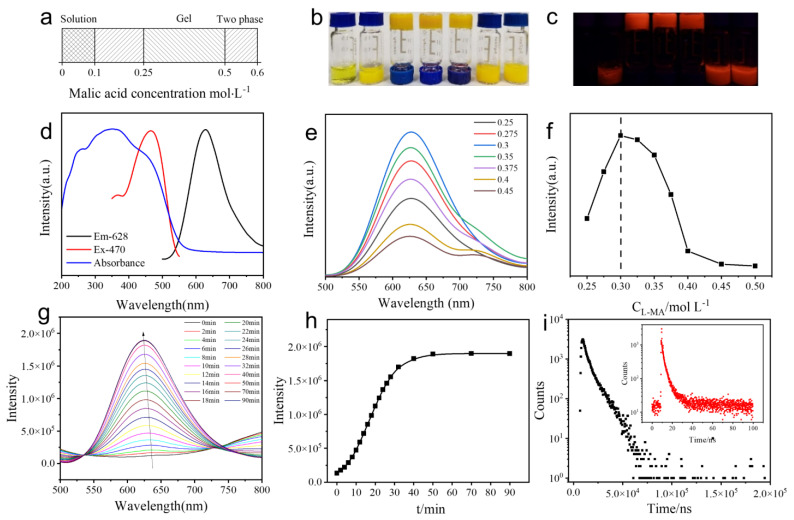
(**a**) Phase transition with different concentration of L–MA. (**b**) Photographs of the hydrogels with different L–MA concentrations. (**c**) Fluorescence photographs of the hydrogels with different L–MA concentrations. (**d**) Photoexcitation (red line, emission = 628 nm), photoemission (black line, excitation = 470 nm) spectra of Ag_9_-NCs hydrogels, and UV–vis absorption (blue line). (**e**) Photoluminescence (PL) spectra of hydrogels with different concentration of L–MA for excitation at 470 nm. (**f**) Comparison of the luminescence intensity of hydrogels with different concentration of L–MA at 628 nm. (**g**) PL spectra of Ag_9_-NCs hydrogels with different incubation times for excitation at 470 nm. (**h**) Comparison of the luminescence intensity of hydrogels with different incubation times. (**i**) PL decay profiles of Ag_9_-NCs hydrogel. Inset: the PL decay profiles of the powder of lyophilized Ag_9_-NCs solution.

The Ag_9_-NCs in an aqueous solution is in a nonfluorescent state. In order to regulate the aggregation behavior and AIE of Ag_9_-NCs, though the non-covalent interaction force dominated by hydrogen bonds, L–MA with hydroxyl and carboxyl functional groups is selected as a hydrogen bond donors, and Ag_9_-NCs, with carboxylate on the periphery, act as hydrogen bond acceptors. Firstly, phase behavior study on Ag_9_-NCs/L–MA mixed system was performed. Based on our previous study [40,41], *c*_Ag9-NCs_ was fixed at 8 mM, while *c*_L–MA_ was changed. It can be seen that different phase behaviors of the samples were obtained (Figure 1a). Compared with the solution without fluorescence (0~0.1 M L–MA) and the sample in the two-phase with weaker fluorescence intensity (0.1~0.25 M L–MA), the hydrogel with higher luminous intensity (0.25~0.5 M L–MA) was selected as the main research object. Once the hydrogels formed, the UV absorption exhibits a broad peak from 200 to 550 nm (Figure 1d) and the electrons of the benzene ring system and C=O undergo π-π* and n-π* transitions, resulting in ultraviolet absorption bands. Moreover, orange–red fluorescence at 628 nm was excited by blue light at 470 nm (Figure 1d) and it can be observed that as *c*_L–MA_ increases, the fluorescence intensity of the hydrogel first increases and then decreases (Figure 1e,f). This is attributed to the fact that when *c*_Ag9-NCs_ = 8 mM and *c*_L–MA_ = 0.3 M, Ag_9_-NCs and L–MA self-assemble to form highly ordered aggregates, and L–MA sufficiently limits the ligands of Ag_9_-NCs, while when *c*_L–MA_ is too high or too low, the uniformity of the formed aggregates is poor, and the self-assembly strategy cannot be effectively implemented, which leads to the weakening of fluorescence intensity. Thus, 8 mM Ag_9_-NCs/0.3 M L–MA hydrogel, which has the highest fluorescence intensity was selected as the typical sample.

Then, the dynamic change of fluorescence intensity with the hydrogel formation was also studied. Once Ag_9_-NCs and L–MA mixed, under the drive of non-covalent interaction forces, the fluorescent intensity of 8 mM Ag_9_-NCs/0.3 M L–MA hydrogel increased immediately within 30 min, achieving AIE effect. After about 1 h, the self-assembly process is almost complete, the fluorescence intensity stabilized and no longer changed (Figure 1g,h). Moreover, the peak position of the fluorescence spectrum has a slight blue shift with time. It is speculated that Ag_9_-NCs, L–MA and water molecules gradually formed the hydrogel through the hydrogen bonds, the vibration of the peripheral ligand of Ag_9_-NCs is restricted, and ligand-to-metal charge transfer occurred, resulting in a blue shift of the peak position. Thus, the AIE phenomenon of Ag_9_-NCs/L–MA hydrogel can be attributed to the limited intramolecular vibration and rotation of the ligand of Ag_9_-NCs, which inhibits the ligand-related non-radiative excited state relaxation and promotes radiation energy transfer. Furthermore, the average of lifetime of Ag_9_-NCs solution is 3.277 ns, while the average of the lifetime of 8 mM Ag_9_-NCs/0.3 M L–MA hydrogel increased to 7.4383 μs (Figure 1i, Table S1), which is approximately 2270 times that of Ag_9_-NCs solution. Therefore, the large Stokes shift (158 nm) and microsecond lifetime (7.4383 μs) indicate that it is essentially phosphors. Besides, the quantum yield of 8 mM Ag_9_-NCs/0.3 M L–MA hydrogel, measured using the integrating sphere, is 11.20%, which is higher than that of aggregates formed by the assembly of Ag_9_-NCs with other substances [42]. Longer fluorescence lifetime and higher quantum yield of Ag_9_-NCs/L–MA hydrogels make it excellent candidates for luminescent sensing materials.

**Figure 2:**
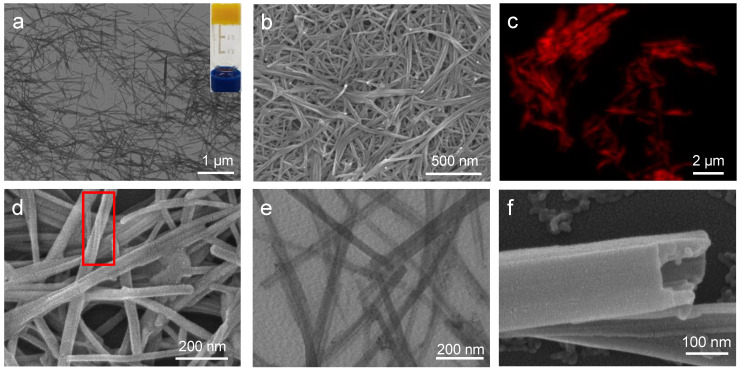
(**a**,**e**) TEM, (**c**) CLSM, and (**b**,**d**,**f**) SEM images of the hollow tubes of 8 mM Ag_9_-NCs/0.3 M L–MA hydrogel with different magnifications (the inset of a is a photograph of the hydrogels).

Scanning electron microscopy (SEM) and transmission electron microscope (TEM) microscopic observations show that the hydrogel is composed of tangled hollow tubes with a very high aspect ratio (20:1), and most of the tubes are between 1–2 μm in length and about 50–100 nm in diameter (Figure 2a–f and Figure S2). The confocal laser scan microscopy (CLSM) image shows that the tubes structure is accompanied by strong fluorescent properties. Further observation by TEM shows that these hollow tubes are composed of a large number of smaller diameter fibers (10–20 nm) (Figure S3). Moreover, it can be seen in SEM that a small amount of hollow tubes are entangled to form a spiral (Figure 2d). It is speculated that the appearance of AIE can be attributed to the appearance of ordered hollow tube structure. The detailed formation mechanism of the hollow-tube structure of Ag_9_-NCs/L–MA hydrogel can be shown in Scheme 1.

### 2.2. Structural Analysis of the Hydrogel

**Figure 3:**
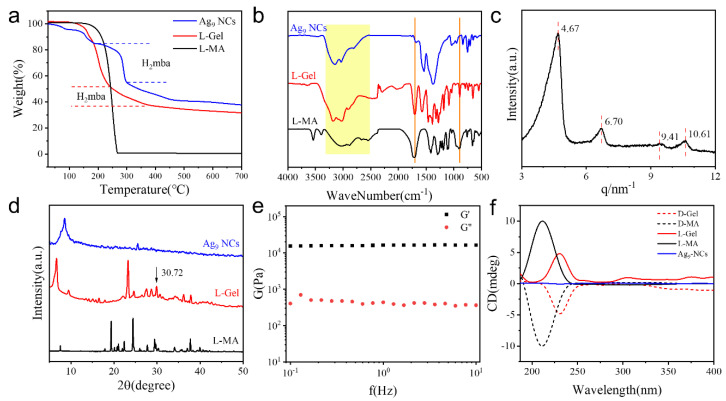
(**a**) TGA and (**b**) FT–IR spectra of lyophilized Ag_9_-NCs solution, L–MA and Ag_9_-NCs/L–MA xerogel. (**c**) SAXS results of Ag_9_-NCs/L–MA xerogel. (**d**) XRD results of lyophilized Ag_9_–NCs solution, L–MA and Ag_9_-NCs/L–MA xerogel. (**e**) Variation of elastic modulus (G′) and viscous modulus (G″) as a function of frequency (τ = 10 Pa). (**f**) CD spectra of Ag_9_–NCs solution, L–MA, Ag_9_-NCs/L–MA hydrogels, D–MA and Ag_9_-NCs/D–MA hydrogels.

In order to further analyze the mechanism of supramolecular self-assembly, a series of characterizations were carried out. Thermogravimetric analyses (TGA) can be used to measure the thermal stability of the studied substances (Figure 3a). The decomposition temperature of L–MA is about 145 °C and the decomposition temperature of the H_2_mba ligand of Ag_9_-NCs is about 200 °C. After assembly, the decomposition temperature of the H_2_mba ligand of Ag_9_-NCs for Ag_9_-NCs/L–MA xerogel is about 260 °C, indicating that the stability of Ag_9_-NCs is further improved by supramolecular self-assembly.

Fourier transform infrared (FT-IR) is an effective tool for analyzing the supramolecular forces (Figure 3b). The broad peak of Ag_9_-NCs/L–MA xerogel at 2500 cm^−1^ to 3200 cm^−1^ represents the stretching vibration of the hydroxyl group, which is stronger and wider than that of Ag_9_-NCs, indicating the formation of hydrogen bonds in the system. The peak at 1700 cm^−1^ representing the stretching vibration of the C=O in the carboxyl group becomes weaker than that of L–MA after the gel is formed, and slightly shifts to a lower wave number, which represents the formation of hydrogen bonds and confirms that L–MA participates in the construction of the hydrogel. The peak at 3540 cm^−1^ represents that the hydroxyl groups in L–MA undergo intermolecular association through hydrogen bonds to form L–MA dimers, which disappear after gelation, indicating that L–MA forms hydrogen bonds with the peripheral ligands of Ag_9_-NCs. The peaks at 1537 cm^−1^ and 1377 cm^−1^ belong to the antisymmetric and symmetric stretching vibration of C=O in carboxylate from ligand of Ag_9_-NCs. For Ag_9_-NCs/L–MA xerogel, the peak of C=O in carboxylate moves, which further confirms the formation of hydrogen bonds between ligand of Ag_9_-NCs and L–MA. Moreover, it can be seen that the absorption peak at 900 cm^−1^, representing O–H in the carboxyl group of L–MA is strong and after the hydrogel is formed, this peak becomes weaker and narrower, confirming that most of the carboxyl groups of L–MA are involved in the formation of hydrogen bonds, limiting the bending vibration of the O–H bonds in the carboxyl groups.

Small- angle X-ray spectroscopy (SAXS) and X-ray diffraction (XRD) can effectively characterize the deposition pattern and spatial structure of the xerogels. SAXS shows that Ag_9_-NCs/L–MA xerogel exhibits four scattering peaks, and the scattering factor q ratio is $1:\sqrt{2}:2:\sqrt{5}$, which is a tetragonal stack (Figure 3c). The smallest repeating unit of the aggregate d = 1.36 nm (d = 2π/q), which is equivalent to the size of Ag_9_-NCs. From the XRD results, it can be observed that L–MA is a triclinic crystal system, while the peak of Ag_9_-NCs/L–MA xerogel at 2θ = 20–40° represents various possible regular arrangements of atomic layers in assembled nanostructures, which is very different from L–MA and Ag_9_-NCs (Figure 3d), indicating Ag_9_-NCs and L–MA formed a multi-complex during the self-assembly. According to the Bragg equation, the interplanar distances of the atomic layer are in the range of 2.37–3.81 Å. Diffraction peaks at 2θ = 30.72 correspond to Ag-Ag, indicating that the d10-d10 argentophilic interaction may exist in the Ag_9_-NCs/L–MA xerogel [40,41,43].

The rheological characteristics are of great significance for supramolecular hydrogels, and the mechanical strength of the Ag_9_-NCs/L–MA hydrogels can be evaluated by the rheological measurement. In the stress scanning (Figure 3e), the storage modulus (G′ = 16,900 Pa) is much larger than the loss modulus (G″ = 614.6 Pa), indicating that the hydrogels exhibit a solid-like nature. The Ag_9_-NCs/L–MA hydrogels exhibit good viscoelasticity, a wider linear viscoelastic region, and a higher yield stress (892.8 Pa), indicating that the hydrogels constructed by the Ag_9_-NCs are more rigid and exhibit strong damage resistance. In the frequency sweep test (Figure S4), G′ is larger than G″ and the moduli of the hydrogels are almost independent of the applied frequency. The above results indicate that the Ag_9_-NCs/L–MA hydrogels have high mechanical strength.

The circular dichroism (CD) spectrum has further confirmed that the Ag_9_-NCs/L–MA hydrogel possesses supramolecular chirality. L–MA has a positive Cotton effect at 212 nm and no obvious CD signal was detected in the Ag_9_-NCs aqueous solution. But the Ag_9_-NCs/L–MA hydrogel has a positive Cotton effect at 230 nm, and the original CD signal of L–MA disappears. Moreover, we also used D-MA to construct Ag_9_-NCs/D-MA hydrogel and it is interesting to find that the CD spectrum of the Ag_9_-NCs/D-MA hydrogel is exactly opposite of the Ag_9_-NCs/L–MA hydrogel. It can be concluded that through supramolecular self-assembly, the molecular chirality of L–MA (or D-MA), was successfully transferred to the supramolecular level, which induced the Ag_9_-NCs/L–MA hydrogel (Ag_9_-NCs/D-MA hydrogel) has supramolecular chirality (Figure 3f).

### 2.3. Ag_9_-NCs/L–MA Xerogel/PMMA Film for Sensing

**Figure 4:**
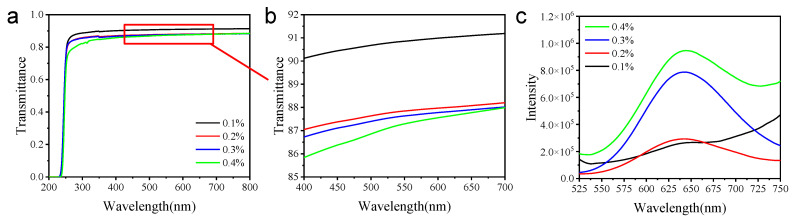
(**a**) transmittance of films with different doping concentrations. (**b**) transmittance of films with different doping concentrations (400–700 nm). (**c**) FL of films with different doping concentrations (from 0.1% to 0.4%).

The composite material obtained by doping the xerogel into the polymer matrix has the advantages of easy processing and good flexibility and can enhance the practical application of the gel [32,33]. PMMA has good UV resistance, chemical durability, and good mechanical properties. Therefore, based on the excellent sensing performance of metal NCs and the advantages of simple preparation of organic films as a matrix material, Ag_9_-NCs/L–MA xerogel are introduced into the organic glass matrix to form a new type of organic film. Herein, the 8 mM Ag_9_-NCs/0.3 M L–MA xerogel is introduced into the PMMA, and through the solvent volatilization method to get a hybrid film. Firstly, several films were prepared with different doping concentration of 8 mM Ag_9_-NCs/0.3 M L–MA xerogel and it can be observed that as the doping concentration increases (from 0.1% to 0.4%), the fluorescence intensity increases but the transmittance decreases (Figure 4a–c). Integrating transmittance and fluorescence spectra, a composite Ag_9_-NCs/L–MA/PMMA film with a mass concentration of 0.3% xerogel was selected to study its sensing performance.

**Figure 5:**
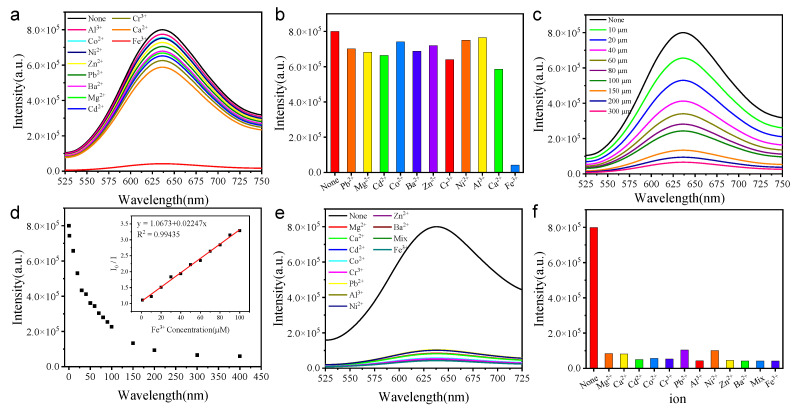
(**a**) Luminescence emission spectra and (**b**) luminescence intensities at 640 nm for 0.3% Ag_9_-NCs/L–MA xerogel/PMMA film in different cations. (**c**) Luminescence emission spectra and (**d**) luminescence intensities at 640 nm for 0.3% Ag_9_-NCs/L–MA xerogel/PMMA film in Fe^3+^ with different concentrations. The insert of d shows Stern–Volmer quenching curve of the luminescence intensity of 0.3% Ag_9_-NCs/L–MA xerogel/PMMA film at 640 nm against Fe^3+^ concentration; (**e**) luminescent emission spectra and (**f**) luminescence intensities at 640 nm for 0.3% Ag_9_-NCs/L–MA xerogel/PMMA film in solutions of different cations with Fe^3+^.

Then, 0.3% Ag_9_-NCs/L–MA xerogel/PMMA films were placed into different metal cation aqueous solutions for 10 min and then, the luminescence spectra of these films were studied (Figures S5 and S6). It is obviously that the composite film shows an obvious quenching effect only toward Fe^3+^ (Figure 5a,b). Furthermore, the 0.3% Ag_9_-NCs/L–MA xerogel/PMMA films were placed into aqueous solutions containing different concentrations of Fe^3+^ ions for 10 min to study the corresponding luminescence intensity (Figure 5c,d). With the gradual increase of *c*_Fe3+_, the luminescence intensities at 628 nm are significantly weakened. In order to investigate the luminescence quenching efficiency, the Stern–Volmer (S-V) equation was used to quantitatively analyze the quenching curve [32]:

(1) $$I_{0}/I=1+K_{SV}[M]$$

where *I*_0_ and *I* are the luminescence intensity at 640 nm when c = 0 and c = *M*, respectively, *K_SV_* is the quenching coefficient of Fe^3+^ ions, and [*M*] is the molar concentration of Fe^3+^ ions. The quenching curve shows a well-fitted linear relationship at low *c*_Fe3+_ (1–100 μM). The *K_SV_* value was calculated to be 2.3 × 10^4^ M^−1^. According to the detection limit expression defined by IUPAC, the detection limit of the composite membrane is calculated to be 0.3 μM, which indicates that the as-prepared 0.3% Ag_9_-NCs/L–MA xerogel/PMMA film material exhibited good sensing performance. The limit of detection of the Ag_9_-NCs/L–MA xerogel/PMMA film far less than the maximum concentration of Fe(III) in drinking water of 5.36 μM (specified by the Minister of Health of the People’s Republic of China).

High sensitivity and high selectivity are the basic requirements of fluorescence sensors. Various mixed metal cation solutions containing Fe^3+^ (1.0 equivalent) and different competing cations (1.0 equivalent) were prepared for competition experiments. By comparing the fluorescence intensity of 0.3% Ag_9_-NCs/L–MA xerogel/PMMA film in different mixed metal cation solutions (Figure 5e,f), it can be seen that the fluorescence is still quenched in the presence of other competing metal cations, which indicates that the composite film has satisfactory selectivity for Fe^3+^ detection. In addition, the used films were picked out and washed with distilled water several times, and the corresponding luminescence intensities cannot be recovered. Therefore, it would be used as a disposable test strip for detection Fe^3+^ ions in drinking water in the future.

**Figure 6:**
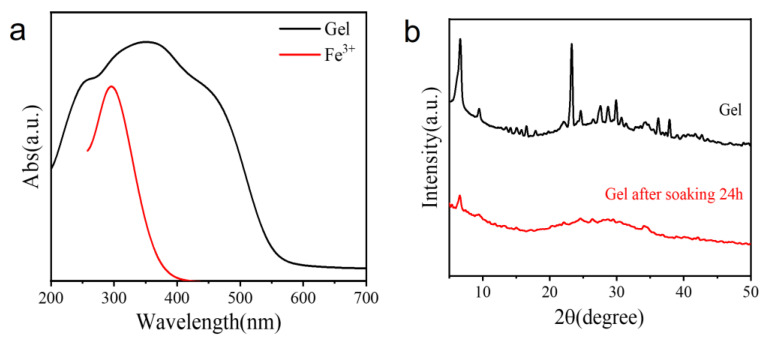
(**a**) UV-Vis absorption of hydrogel and Fe^3+^. (**b**) XRD of xerogel and xerogel after Fe^3+^ soaking 24 h.

The possible sensing mechanism of Fe^3+^ ions quenching the fluorescence of 0.3% Ag_9_-NCs/L–MA xerogel/PMMA film was further analyzed. It can be seen from UV-Vis (Figure 6a) that the absorption peaks of Fe^3+^ and the Ag_9_-NCs/L–MA hydrogel overlap in the absorption range, indicating that the competitive absorption of Fe^3+^ reduces the energy transfer efficiency [42]. Moreover, structural collapse or change maybe also cause the luminescence to be quenched [32]. The XRD test was performed on the Ag_9_-NCs/L–MA xerogel before and after immersion in Fe^3+^ ion aqueous solution (Figure 6b). The XRD spectra of the samples were not consistent, which means that the structure of the Ag_9_-NCs/L–MA xerogel was destroyed after treated by Fe^3+^ ion aqueous solution. Therefore, the collapse of the crystal structure is also one of the reasons for the quenching of luminescence. Thus, it can be concluded that the fluorescence quenching of Ag_9_-NCs/L–MA xerogel/PMMA film is caused by the competitive absorption of Fe^3+^ and the destruction of the crystal structure. The fluorescence quenching phenomenon of the Ag_9_-NCs/L–MA xerogel/PMMA film is shown in Scheme 1.

## 3. Conclusions

In summary, through self-assembly, Ag_9_-NCs, and L–MA formed a supramolecular hydrogel, with highly ordered aggregates, by regulating a variety of non-covalent interactions. The nanostructure (tangled hollow tubes) in the hydrogel indicated that L–MA restricts the intramolecular vibration and rotation of the ligand of the Ag_9_-NCs, so that it can emit a stable and bright orange–red phosphorescent emission. The excellent photoluminescence properties of Ag_9_-NCs/L–MA xerogel make it likely to be used as highly sensitive probes for Fe^3+^. The Ag_9_-NCs/L–MA xerogel/PMMA composite film can selectively identify Fe^3+^, the quenching coefficient *K_SV_* is 2.3 × 10^4^ M^−1^, and the detection limit is 0.3 μM. The composite film also possesses good recyclability and anti-interference ability, with respect to another ions. The current research aims to achieve AIE, by precisely regulating the formation of ordered aggregates of metal NCs, broadening the research field of metal NCs, and enriching the practical applications of these luminescent materials.

## 4. Experiment Section

### 4.1. Materials

Ag_9_-NCs was synthesized and purified, according to our previous work, which have a crystal structure [44]. L–MA and D-MA were purchased from Sinopharm Chemical Reagent Co (Shanghai, China) and used without further purification. Ultrapure water used in the experiments, with a resistivity of 18.25 MΩ cm^−1^, was obtained using a UPH-IV ultrapure water purifier (Sichuan, China). Polymethylmethacrylate (PMMA, average Mw: ~350,000) was purchased from Sigma-Aldrich (Shanghai, China). Dichloromethane (CH_2_Cl_2_) were obtained from local supplier with the quality of analytical grade and used without further purification.

### 4.2. Synthesis of Ag_9_-NCs/L–MA Hybrid Nanostructures

In this experiment, 0.5 mL of Ag_9_-NCs solution (15.87 mM) was added to 0.5 mL L–MA solution (0.6 M) with stirring. The hydrogel was successfully prepared after 8 h of constant temperature (20 °C) in a thermostat. The hydrogels was lyophilized in a vacuum extractor at 60 °C for 5 day to collect the orange–yellow powder.

### 4.3. Fabrication of Ag_9_-NCs/L–MA/PMMA Composite Thin Film

The PMMA powder (200 mg) was dissolved in dichloromethane (8 mL), then followed by addition of the corresponding required of orange–yellow Ag_9_-NCs/L–MA xerogels. After being evenly dispersed, place it in a petri dish with a diameter of 6 cm at room temperature for 5 h. Each set of data has been measured 3 times using different batches of film to reduce the error.

### 4.4. Characterization

A copper mesh was inserted into the gel to obtain a sample and, after drying under an IR lamp for 45 min, TEM images were observed under a JCR-100CX II (JEOL) microscope. The gel was placed on a silica wafer, dried for 45 min under an IR lamp, and observed by field-emission SEM and AFM, respectively. UV−vis data were recorded on a Shimadzu UV-2600 spectrophotometer. Fluorescence data were tested on an LS-55 spectrofluorometer (PerkinElmer, Waltham, MA, USA) and an Edinburgh Instruments FLS920 luminescence spectrometer (xenon lamp, 450 W), respectively. SAXS measurements were performed using an Anton-Paar SAX Sess mc^2^ system with nickel-filtered Cu Kα radiation (1.54 Å) operating at 50 kV and 40 mA. XRD patterns were taken on a D8 ADVANCE (Germany Bruker) diffractometer, equipped with Cu Kα radiation and a graphite monochromator. FT-IR spectra in KBr wafer were recorded on a VERTEX-70/70v spectrophotometer. CLSM observations were performed using an inverted microscope (model IX81, Olympus, Tokyo, Japan), equipped with a high-numerical-aperture 60 oil-immersed objective lens. The rheological measurements were carried out on an Anton-Paar Physica MCR302 rheometer with a cone–plate system. Before the frequency sweep, an amplitude sweep at a fixed frequency of 1 Hz was carried out to ensure that the selected stress was in the linear viscoelastic region. The frequency sweep was carried out from 0.01 to 100 Hz at a fixed stress of 10 Pa. TGA was performed under a nitrogen atmosphere at 25–700 °C, with a heating speed of 10 °C min^−1^ on a TA SDT Q600 thermal analyzer. CD spectra were obtained using a JASCO J-810 spectropolarimeter, which was flushed with nitrogen during operation. The absolute fluorescence quantum yields were measured with a spectrofluorometer (FLSP920, Edinburgh Instruments Ltd., Livingston, UK), equipped with an integrating sphere.

## Supplementary Materials

The following are available online at https://www.mdpi.com/article/10.3390/gels7040192/s1, Figure S1: The molecular structure of Ag_9_-NCs, Figure S2: AFM image of fibers, Figure S3: (a–b) TEM image of the fibers. (c–d) SEM image of fibers, Figure S4: Elastic modulus (G′) and viscous modulus (G″) as a function of the applied stress at a constant frequency (1.0 Hz), Figure S5: Photographs of Ag_9_-NCs/L–MA xerogel/PMMA film under different conditions, Figure S6: Luminescence emission spectra of 0.3% Ag_9_-NCs/L–MA xerogel/PMMA film in 100 μM Fe^3+^ aqueous solution with different interaction time, Table S1: The average lifetime of Ag_9_-NCs and hydrogel.
