# Supplementary material for: Luminescent Hydrogel Based on Silver Nanocluster/Malic Acid and Its Composite Film for Highly Sensitive Detection of Fe3+
Source: Gels. 2021 Oct 31;7(4):192. doi: 10.3390/gels7040192 (PMC8628787; doi:10.3390/gels7040192)
Supplement: Supplementary file 1 [file gels-07-00192-s001.zip › gels-1419311-supplementary.pdf]

Supporting Information

Luminescent Hydrogel Based on Silver Nanocluster/Malic Acid and Its Composite Film for Highly Sensitive Detection of Fe^{3+}

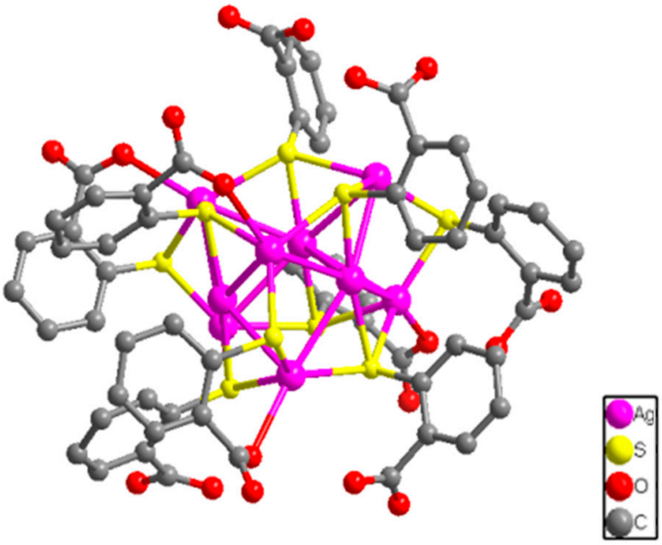

Figure S1. The molecular structure of Ag_9-NCs.

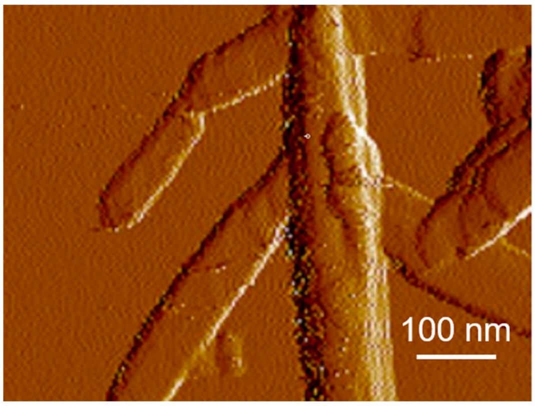

Figure S2. AFM image of fibers.

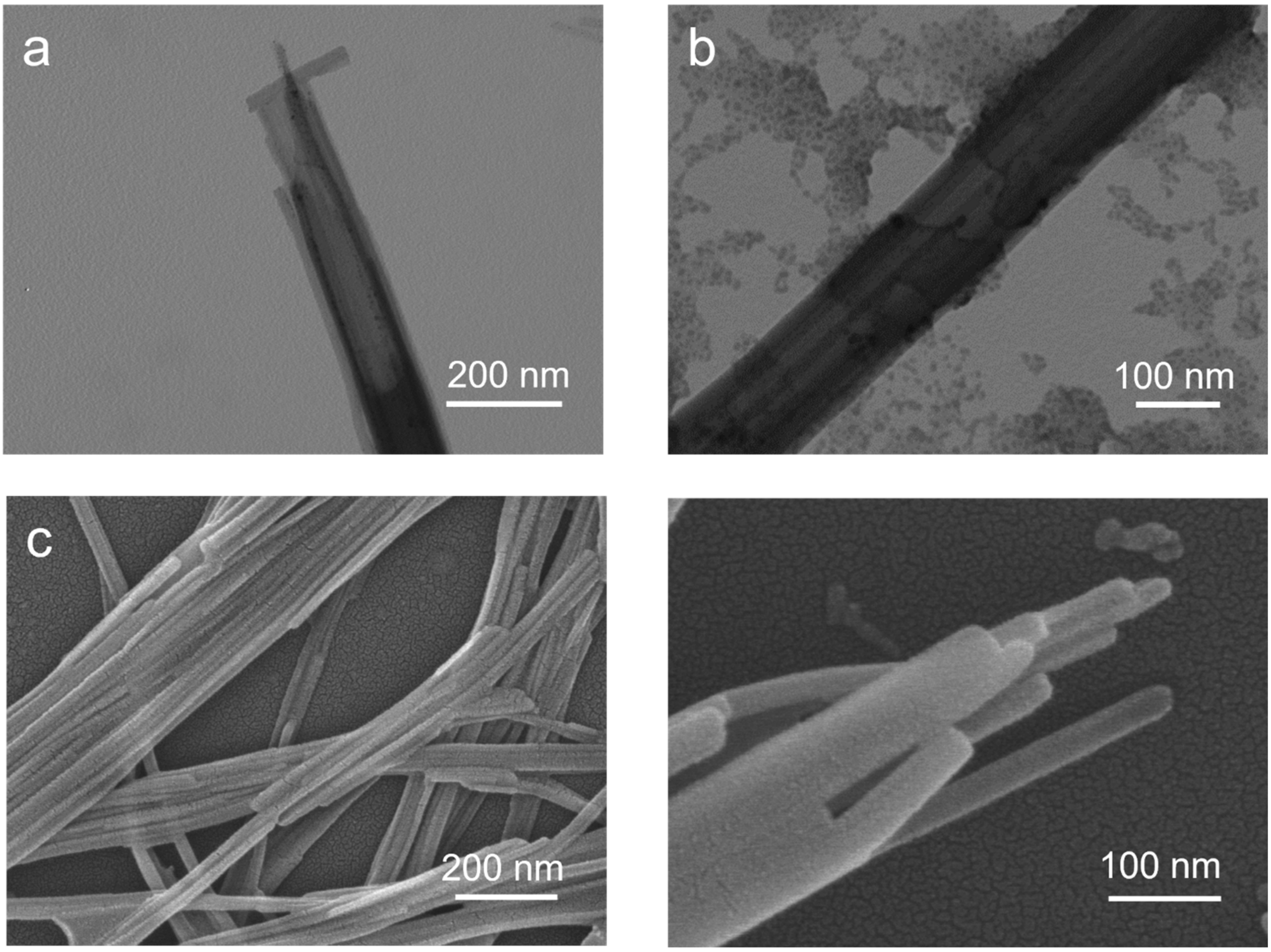

Figure S3. (**a**–**b**) TEM image of the fibers. (**c**–**d**) SEM image of fibers.

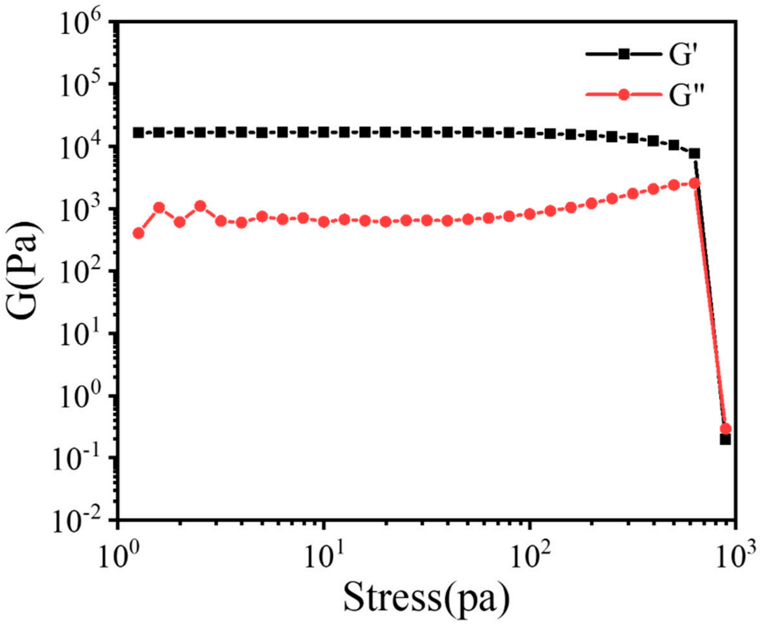

Figure S4. Elastic modulus (G') and viscous modulus (G'') as a function of the applied stress at a constant frequency (1.0 Hz).

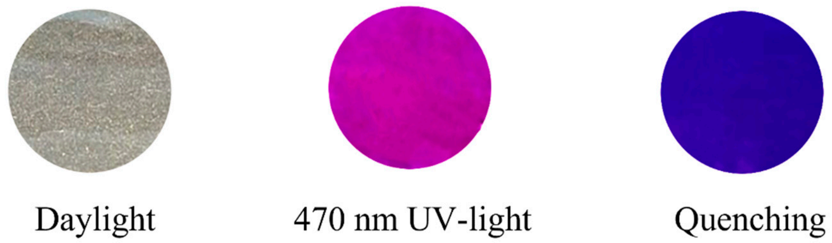

Figure S5. Photographs of Ag_9-NCs/L-MA xerogel/PMMA film under different conditions.

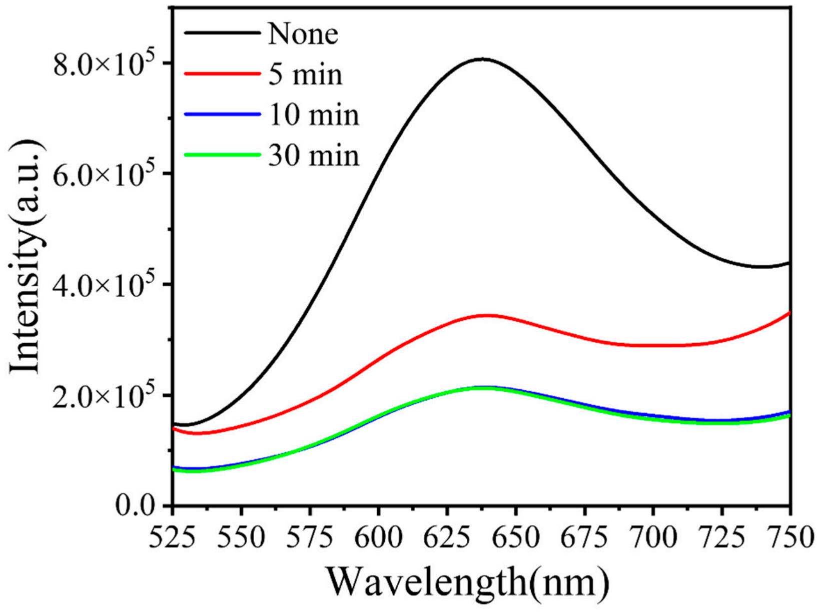

Figure S6. Luminescence emission spectra of 0.3% Ag_9-NCs/L-MA xerogel/PMMA film in 100 μM Fe^{3+} aqueous solution with different interaction time.

Table S1. The average lifetime of Ag_9 NCs and hydrogel.

**Samples/Lifetime**	**τ_1/ns**	**τ_2/ns**	**τ_3/ns**	**τ_{ave}/ns**
Ag_9-NCs	0.032(13.1%)	1.973(50.7%)	6.283(36.2%)	3.277
Hydrogel	2800.9(33.53%)	9777.6(66.47%)	—	7438.3
